# Hashimoto’s Thyroiditis and Papillary Thyroid Carcinoma: A Follow-Up Study in Patients with Absence of Aggressive Risk Factors at the Surgery of the Primary Tumor

**DOI:** 10.3390/diagnostics13193068

**Published:** 2023-09-27

**Authors:** Andrea Marongiu, Susanna Nuvoli, Andrea De Vito, Sonia Vargiu, Angela Spanu, Giuseppe Madeddu

**Affiliations:** 1 Unit of Nuclear Medicine, Department of Medicine, Surgery and Pharmacy, University of Sassari, 07100 Sassari, Italy; snuvoli@uniss.it (S.N.); sovargiu@tiscali.it (S.V.); aspanu@uniss.it (A.S.); giuseppe.madeddu@email.it (G.M.); 2 Unit of Infectious Diseases, Department of Medicine, Surgery and Pharmacy, University of Sassari, 07100 Sassari, Italy; andreadevitoaho@gmail.com

**Keywords:** Hashimoto thyroiditis, papillary carcinoma, follow-up, neck lymph node metastasis, distant metastasis, risk factors, ^131^I SPECT/CT, thyroglobulin, thyroglobulin antibody, thyroid peroxidase antibody

## Abstract

Hashimoto’s thyroiditis (HT) is often associated with papillary thyroid carcinoma (PC); it is still a matter of controversy whether the behavior of carcinoma is more aggressive or not. During the follow-up, we retrospectively enrolled 97 patients with PC/HT after thyroidectomy without risk factors at the surgery of the primary tumor, such as multifocality/multicentricity, extrathyroid tumor extension, vascular invasion, neck and distant metastases, and aggressive histological variants. HT diagnosis was confirmed by histology and serum thyroid antibodies. Tumor size was ≤10 mm in 64 cases (microcarcinomas); 206 matched PC patients after thyroidectomy without HT and risk factors were enrolled as controls, totaling 122 microcarcinomas. During follow-up, metastases occurred in 15/97 (15.5%) PC/HT cases, eight microcarcinomas, and in 16/206 (7.8%) without HT, eight microcarcinomas (*p* = 0.04). Considering both PC/HT and PC patients without HT who developed metastases, univariate analysis showed an increased risk of metastases in patients with HT coexistence, OR: 2.17 (95% CI 1.03–4.60) *p* = 0.043. Disease-free survival (DFS) was significantly (*p* = 0.0253) shorter in PC/HT than in the controls. The present study seems to demonstrate that HT is not a cancer protective factor in PC patients given the less favorable outcomes and significantly shorter DFS. HT may also represent an independent recurrence predictor without other risk factors.

## 1. Introduction

An increased risk of developing differentiated thyroid carcinoma (DTC) and particular papillary carcinoma (PC) has often been reported in patients with chronic Hashimoto’s thyroiditis (HT), which is an autoimmune inflammatory disease in which the immune system reacts against some thyroid agents and represents the most common disorder that leads to hypothyroidism [1]. HT pathology consists of diffuse lymphoplasmacytic infiltration, fibrosis, and parenchyma atrophy; however, germinal centers, enlarged epithelial cells with large nuclei, and eosinophilic cytoplasm may be present.

Dailey first described the coexistence of HT and PC [2]. Some later studies reported in the literature have observed that the association of PC and HT ranged from 5 to 85% [3] and from 10 to 58% [4], with an elevated risk of PC in patients with HT [5,6,7]. The probability of this coexistence tends to increase in males under 40 years old [6].

However, the exact pathogenesis of the relationship between the two associated disorders appears unclear and it is a matter of debate [8,9].

It also remains controversial that the inflammatory disease may affect the outcome of PC/HT patients after thyroidectomy and radioiodine ablation, with a particular interest in the prognosis of PC.

Some authors have observed that PC with associated HT presents a favorable prognosis [3,8,10,11,12,13,14,15,16], and the presence of HT may be considered an immune reaction to control tumor growth and proliferation [13]. Moreover, HT may also be protective against tumor progression [12,17,18]. However, according to other authors [19,20], despite having reported an improved prognosis in patients with HT associated with PC, HT was not associated with a lower recurrence rate or a lower frequency of distant metastases [19]. At the same time, although HT was associated with a minor risk of recurrence during long-term follow-up, this result did not appear significant when other good prognostic factors were added, such as younger ages and female sex [20]. In further studies, it has also been observed that HT may have only a minimal impact on the development of the tumor and may not modify its aggressiveness [9]. For others, PC and chronic thyroiditis coexistence might be the unfavorable factor of PC in patients <55 years and the predictor of higher recurrence risk [21].

Furthermore, the significance of the coexistence of PC and HT with neck lymph node metastasis during follow-up continues to be debated. Some studies reported that there was not any difference in the prevalence of central and lateral lymph node metastases between patients with and without HT [22]. In other studies, a low incidence of lymph node metastases in PC patients with concurrent HT was observed at the surgery of the primary tumor [23], as well as a reduced incidence of lymph nodes of the central compartment, and a lower number of lesions with respect to PC patients without HT was ascertained [24,25,26,27,28,29]. Moreover, a lower rate of recurrences and longer disease-free survival (DFS) was also reported [3,8,10,11,12,13,20]. However, other studies [30,31] did not confirm these data, observing a more elevated spread of PC to the lymph nodes of the central compartment with respect to PC cases without HT (76.4% vs. 22.8%; *p* < 0.001). Furthermore, in other studies, the presence of HT was reported to be correlated with a less aggressive PTC, thus suggesting a better prognosis, but it did not evidence a reduced recurrence rate and frequency of distant metastases and a longer DFS [29]. Still, others did not observe a significant difference in overall survival (OS) in patients with HT [15].

This present study aimed to assess the influence of HT on the progression of PC during follow-up after total thyroidectomy and radioiodine ablation in a group of HT/PC patients with the absence of risk factors at the surgery of the primary tumor. The latter consists of extrathyroid tumor extension, both extended (eETE) and minimal (mETE), vascular invasion, multifocality/multicentricity, neck lymph nodes (in both the central compartment and the lateral cervical neck regions) and distant metastases, and aggressive histological variants. This HT/PC group of patients was compared with matched sex/age PC patients without HT and with none of the risk factors, as above.

## 2. Materials and Methods

### 2.1. Patients

Among a large group of patients with HT and associated PC, 97 consecutive patients were enrolled retrospectively in the course of follow-up after total thyroidectomy and ablation with 131-iodine (group 1). The patients were characterized by the absence of risk factors at the surgery of the primary carcinoma, including extrathyroid tumor extension (extended and minimal ETE), vascular invasion, multifocality/multicentricity, neck lymph node (in both the central compartment and the lateral cervical neck regions) and distant metastases, and aggressive histological variants. Some of these patients underwent surgery for the presence of nodules suspected of cancer at biopsy and others for multinodular goiter, all of them with pictures of chronic inflammation at the ultrasound, thyroglobulin antibody (AbTg), and thyroid peroxidase antibody (AbTPO) positivity by probable Hashimoto thyroiditis, which was confirmed at histology after surgery.

Moreover, a group of 206 PC patients without HT confirmed at surgery, who served as the controls, was also enrolled (group 2). In the latter cases, surgical treatment was indicated when suspect lesions of cancer were detected in the FNAB of thyroid nodules or multinodular goiters. In none of these, histology ascertained a condition of chronic lymphocytic thyroiditis; serum AbTg and AbTPO were also absent.

In all patients of the two groups, total thyroidectomy was performed according to the therapeutic strategy of choice in the University Surgery Department and in no patient were post-operative complications observed, such as recurrent laryngeal nerve invasion or hypocalcemia, etc.

The central compartment neck dissection was performed in 32 patients and laterocervical neck lymph node dissection in was performed 12 cases. The latter was removed when suspected by imaging before surgery but did not reveal neoplastic lesions.

The characteristics of the patients at the surgery of the primary tumors, according to the 8th Edition AJCC Cancer Staging [32], are reported in Table 1.

None of the patients with HT of group 1 were on thyroid hormone therapy since they had no clinical or subclinical hypothyroidism conditions before surgery.

Exclusion criteria for PC/HT were applied at the surgery of the primary tumor when the patients were in hypothyroidism condition and were treated with thyroid hormone replacement. Moreover, the patients were also excluded when clinical and laboratory thyroid function tests, nuclear medicine, and radiologic procedures were characteristic for Graves’ disease with the presence of TSH receptor antibodies (TRAbs) before the surgery and when those risk factors previously mentioned were present at histology.

The carcinomas of the 206 patients of group 2 were single nodules in 179/206 cases and were included in an MNG in 27/206 cases; these latter ones were only detected during the surgery or in histological section exams post-operation.

Like PC/HT, no PC control patients showed the previously mentioned risk factors at the surgery of the primary tumor. Moreover, before surgery, they had normal thyroid function tests and none of them had been treated with L-thyroxine to reduce the growth of single nodule or multinodular goiter.

No statistical difference exists for the two groups of patients (PC/HT and controls) regarding all the already mentioned parameters illustrated in Table 1.

A diagnosis of HT was made in all cases by histological specimens removed during surgery, and it was based on the presence of widespread infiltrations of lymphocytes and plasma cells, oxyphilic cells, and lymphoid follicles or germinal centers in the completely normal gland. However, peri-tumoral lymphocytic infiltration was not considered HT, since this may reflect an immune response to the carcinoma; the cases with only focal lymphocytic infiltration areas were similarly excluded.

The diagnosis of HT was also confirmed by the presence of thyroid antibodies, AbTg, and/or AbTPO over cut-off.

The distribution of antithyroid antibodies is shown in Table 2.

PC/HT and PC patients without HT were followed in a long-term follow-up after thyroidectomy and radioiodine therapy with a mean period of 99.09 ± 32.21 months. In particular, PC/HT patients were followed up for 103.7 ± 28.08 months and control patients for 97.12 ± 33.4 months, both of them between 2006 and 2022; the difference is not statistically significant (*p* = 0.374).

In the case of suspected metastases during follow-up, the diagnosis was confirmed by histology. However, in some cases, the lack of histopathological findings of the suspect lesions was due to the difficulty of identifying the potential site of the metastases. Thus, when histology was not available, the diagnosis was confirmed by a close follow-up with clinical and imaging examinations and sequential evaluation of thyroglobulin serum level changes for at least 18 months and over 120 months.

### 2.2. Methods

During follow-up, the patients were monitored with different diagnostic procedures, such as clinical exams, ultrasound, ^131^I whole-body scanning (WBS), and ^131^I single-photon emission computerized tomography/computerized tomography (SPECT/CT). The latter two were performed 24–48 and 72 h, respectively, after 185 MBq radioiodine diagnostic dose using a hybrid dual-head gamma camera in patients with hypothyroidism after L-thyroxine withdrawal or after recombinant hormone (rh) thyroid-stimulating hormone (TSH) with serum TSH levels always over 50 µU/mL. If necessary, CT, PET/CT, and needle biopsy by ultrasound were also performed, the latter when the lesions were only easily accessible.

Sequential measurements of serum thyroglobulin and AbTg and AbTPO were also measured; these were assayed by the immunoassay method using chemiluminescence. The assay detection limit of thyroglobulin is 0.1 ng/mL. For thyroglobulin positivity, the cut-off was considered to be <0.2 ng/mL during suppressive therapy and <1 ng/mL after rh-TSH stimulation. The AbTg cut-off was 100 IU/mL and the AbTPO cut-off was 16 IU/mL.

In the University Hospital Nuclear Medicine Center, where the present study was conducted, all nuclear medicine examinations were performed. Four nuclear medicine physicians (G.M, A.M., S.N., and A.S.) were aware of the reason for the exams but unaware of the data from other studies conducted previously.

There was very low inter-observer variability, and discrepancies were resolved through consensus.

### 2.3. Statistics

To assess the normality of quantitative data, the Shapiro–Wilk test was used. Quantitative variables were summarized with mean ± standard deviation (SD) or medians and 25–75° percentiles (IQR), whereas qualitative ones were summarized by absolute and relative (percentages) frequencies. The Mann–Whitney test or Student’s *t*-test evaluated the quantitative variables’ subgroup differences. To assess differences in qualitative variables, Pearson’s chi-squared or Fisher’s exact tests was employed. Fisher’s chi-squared test was used to evaluate categorical variables.

To test the association between the collected variables and the risk of metastases, logistic regression analysis was used. The multivariate logistic regression included independent variables, resulting in *p* < 0.10 in the univariate analysis. Metastases were considered a dependent variable. The significance level was defined as *p* < 0.05. To assess the 10-year disease-free survival visually, Kaplan–Meier curves were plotted, employing the log-rank test to assess the statistical difference between patients with HT and patients without HT. A two-tailed *p* < 0.05 was considered statistically significant. All statistical analyses were performed with STATA version 16.1 (StataCorp. LLC, College Station, TX, USA).

## 3. Results

Among the 303 patients enrolled in the present study, namely 97 with PC/HT (group 1) and 206 PC without HT (group 2), 31 cases (10.2%) developed metastases during the long-term follow-up. Of these, 15/97 (15.5%) belonged to group 1, and 16/206 (7.8%) belonged to group 2. The difference was statistically significant (*p* = 0.04). Tumor recurrences were defined when neck lymph nodes and distant metastases appeared in a period ≥18 months after surgery and radioiodine ablation.

The results of 15 PC/HT patients who developed metastases (group 1a) are reported in Table 3. A total of 8/15 (53.3%) of these cases had microcarcinoma (2 ≤ 5 and 6 > 5 mm).

In the other 82/97 PC/HT patients (group 1b) who did not develop metastases during follow-up (not entered in the table), 75 cases had a classic variant of PC and 7 a follicular variant, 56 of whom were classified as microcarcinomas, 31 (55.4%) ≤5 mm and 25 (44.6%) >5 mm.

Thyroglobulin levels at the surgery of the primary tumor were undetectable in 51 patients and over the cut-off in the remaining 31 cases. Thyroid antibodies, AbTg, and/or AbTPO were over the cut-off in 65/82 cases.

In patients of group 1b (not entered in the table), thyroglobulin was undetectable or very low (≤1 ng/mL) during the long-term follow-up, and thyroid antibodies, AbTg, and AbTPO were absent in all cases, except in 10/82 patients in whom antibodies were over the cut-off.

In particular, AbTg levels were significantly (*p* = 0.0379) more elevated in the patients of group 1a [1116.37 ± 3166.88 IU/mL (median 153.6)] with respect to those of group 1b [274.72 ± 758.83 IU/mL (median 63)], as well as AbTPO [125.99 ± 184.64 IU/mL (median 57) vs. 20.445 ± 32.98 IU/mL (median 10); *p* = 0.00048].

Among the 206 PC patients without HT (group 2), considered as the controls, 16 of whom developed metastases during long-term follow-up, a classic variant of PC was found at histology in 15 cases and a follicular variant was found in the remaining case; 8 of them were microcarcinomas, 3 ≤ 5 mm and 5 > 5 mm (Table 4).

Of the other 190 patients of group 2 (not entered in table) who did not develop metastases (group 2b), 164 had a classic variant of PC, and 26 had a follicular variant; 114 of these had a microcarcinoma (54 ≤ 5 mm and 60 > 5 mm). All patients had thyroglobulin levels under 0.2 ng/mL in hypothyroidism condition or <1 ng/mL after rh-TSH stimulation, and AbTg/AbTPO levels were undetectable or under the cut-off.

As shown in Table 5, at univariate analysis, only HT showed that there was an increased risk of metastasis appearance for patients with HT during the follow-up (OR: 2.17 (95% CI 1.03–4.60) *p* = 0.043).

Furthermore, in the patients with HT, metastases appeared in a shorter time than in the patients without HT; the difference between median values of both groups was not statistically significant [25 (IQR 23–48) vs. 28 (IQR 24.5–44) months, *p* = 0.24].

**Figure 1:**
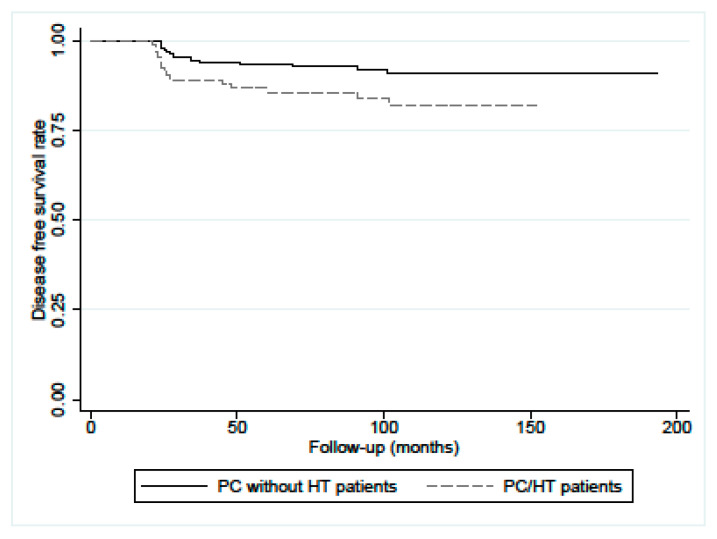
Disease-free survival (DFS) in PC/HT patients (n.97) and matched PC control patients without HT (n.206). The difference, calculated with the log-rank test, was statistically significant (*p* = 0.0253).

Evaluating the disease-free survival (DFS) at 10 years, this was significantly lower in the PC/HT patients than the patients without HT (81% vs. 91%, *p* = 0.0253), as shown in Figure 1.

**Figure 2:**
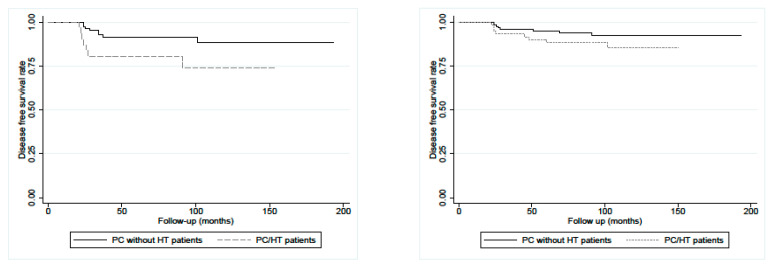
At **left**: Disease-free survival (DFS) in PC/HT patients (n.33) and matched PC control patients without HT (n.84) with carcinoma >10 mm showed a statistical difference (*p* = 0.044, calculated with log-rank test). At **right**: Disease-free survival (DFS) in PC/HT patients (n.64) and matched PC control patients without HT (n.122) with microcarcinoma (≤10 mm) did not show statistical difference (*p* = 0.159, calculated with log-rank test).

Going into more detail, the results show that the significance (*p* = 0.044) was present when considering the patients with a size of carcinoma >10 mm. However, in patients with microcarcinomas (tumor size ≤10 mm), the difference was not statistically significant (*p* = 0.159) as illustrated in Figure 2.

## 4. Discussion

Thyroid autoimmune diseases were often associated with differentiated carcinoma and particularly with PC. Both Graves’ disease (GD) and HT can influence the growth and the progression of carcinomas, affecting the prognosis and the outcome of the affected patients. However, it is a matter of controversy whether the behavior of the carcinomas is more aggressive when the two diseases are associated with thyroid cancer. It has been observed that the appearance of metastatic lesions during follow-up is more frequent in patients with PC/GD [34,35,36,37] and PC/HT [21,22,30,31] than in PC patients without these autoimmune diseases. However, other studies have observed no difference in the outcome in the patients with or without GD and HT [15,38,39], while others have suggested that the two diseases may be factors of a more favorable disease course [3,8,10,11,12,13,20,40,41]. Thus, the prognosis of PC with associated HT continues to remain an active focus of debate.

Regarding the pathogenesis of HT associated with PC, a possible cause can be referred to as the infiltration of lymphocytes and other sensing cells, cytokines, chemokines, and growth factors. All of these are components of cellular neoplastic transformation and tumor progression [42,43,44]. Moreover, other hypotheses have been proposed, such as the active inflammatory response present in patients with HT that can create a favorable setting for malignant transformation with DNA damage through the formation of reactive oxygen species (ROS), resulting in mutations that eventually lead to the development of PC [45]. Anyway, the relationship between inflammation and PTC is complex and still not completely understood.

The current debated issue of the relationship between HT and PC has further been evaluated in the present, retrospective study, mainly the behavior of carcinoma associated with HT during a long-term follow-up after thyroidectomy and ^131^I ablation.

Moreover, a group of PC patients without HT was also enrolled, as controls, in whom the thyroid autoimmune disease had been excluded both at histology and in the serum and had been operated on during the same period of PC/HT; they were similarly exempt from the same risk factors, as above.

At the surgery of the primary tumor, PC/HT patients were mainly associated with younger age, more females, and smaller tumor size with respect to PC without HT cases, but not in a statistically different way. As previously mentioned, no PC patient with or without HT had a clinical or subclinical hypothyroidism condition with high TSH levels and none were on L-thyroxine replacement. This aspect is important since HT patients with associated PC may develop hypothyroidism with an increase in TSH serum levels, which can be responsible for a high frequency of PC [4,46,47]. L-thyroxine therapy, by reducing serum TSH levels, can slow the progression of the tumor, reducing the frequency of recurrences and cancer-related mortality rate [46,47]. During the follow-up, a significantly higher rate of disease progression was observed in PC/HT patients compared to PC-matched control patients without HT, in spite of similar tumor characteristics. The percentage of lymph node metastases was significantly higher in patients with HT than in the control cases. However, the development of distant metastases only occurred in one of the control cases. Moreover, DFS at ten years was significantly shorter in PC/HT patients than in the cases without HT. In addition, in most PC/HT patients, neck and distant metastases appeared in a shorter period after thyroidectomy and radioiodine ablation with respect to the controls, but the difference was not statistically significant.

Metastases from DTC after thyroidectomy and radioiodine ablation have usually been identified during long-term follow-up using traditional diagnostic imaging procedures. Among these, ultrasound was employed and sometimes associated with fine-needle aspiration biopsy (FNAB), as well as nuclear medicine exams, and in particular, ^131^I-SPECT/CT, more recently applied in the clinical field [48,49,50,51,52], and sequential serum thyroglobulin and AbTg assays in the two groups of patients and AbTPO in PC/HT cases. In general, CT and MRI, and when the carcinomas are poorly differentiated by ^18^F-FDG PET/CT [53], represented the other diagnostic imaging highly sophisticated procedures.

Moreover, in PC/HT patients who developed metastases, it may not be excluded that HT alone could represent an independent predictive risk factor for metastasis appearance with the worsening of the prognosis of the disease and unfavorable outcomes. The calculation that confirms this result has been achieved using univariate analysis comparing PC/HT and PC without HT.

The results of the present study seem to demonstrate that PC associated with HT proved more aggressive than PC without HT but with no correlation with cancer mortality rate compared to the controls. Regarding this, none of the PC/HT patients died because of the thyroid tumor during the period of study, similar to what was observed in the control patients.

The results obtained in this study related to the aggressiveness of PC in patients with HT were also observed by other authors [21,30,31]. Among these, several pathologic features of aggressiveness have also been observed in a study conducted of patients with HT associated with PC in areas of endemic goiter with iodine deficiency, in which the PC variant constituted 74.1% of thyroid cancer [54]. The same authors hypothesized that this condition might portend aggressive behavior. However, in this casuistry, many patients with aggressive histological variants of PC were present. Anyway, the pathologic features observed in PC associated with HT might be attributed, at least in part, to the endemic nature of the area of iodine deficiency of origin of patients. These results, in the opinion of the authors, seem to suggest a wider interaction between iodine supply, autoimmunity, and carcinogenesis.

However, the above data were in contrast to what was obtained by others in several studies that reported an excellent prognosis and favorable disease-free survival of the patients also considering HT association with PC as a protective factor against tumor progression [3,8,10,11,12,13,14,15,16,17,18,20]. Furthermore, the metastatic LN ratio, defined as the number of metastatic LNs divided by the number of removed LNs, was smaller in PC/HT patients with respect to PC cases without HT [16,22], and the recurrence rate in the patients with HT (1.5%) was also significantly (*p* < 0.042) lower than that in the cases without HT (5.0%) [22]. However, the intimate cause of these conflicting results is still unclear.

Most PC/HT patients who developed metastases were females aged ≤55 years and with a size of carcinoma ≤10 mm (microcarcinoma). These patients, in a higher percentage, were represented by cases with carcinomas ≥5 mm and did not show any statistical significance in DFS with respect to PC without patients with HT, unlike PC/HT patients with carcinoma >10 mm.

As is well known, papillary microcarcinoma (PTMC) presents minimal invasiveness and excellent long-term prognosis; however, PTMC, in some cases, can be more aggressive with multifocal and bilateral involvement, extracapsular extension, and neck lymph node and distant metastases [55,56,57]. Thus, PTMC identification is mandatory for the earliest treatment, and long surveillance after surgery in the follow-up is justified, considering that the recurrence rate extends for many years, as also confirmed in previous studies on PTMC [57]. A higher appearance of cervical LN metastases has been found with respect to PC/HT patients with microcarcinoma and with respect to PC patients without HT, although without statistical significance [58]. However, despite that, the presence of HT did not seem a worse prognostic factor, but the authors, because of the high probability of cervical lymph node metastases, maintained that underlying HT might be considered a risk factor for neck metastases in PTMC patients. Moreover, another study has suggested that coexistent HT in patients with PTMC had an insignificant protective effect on lymph node metastases and prognosis, whose incidence was similar between patients with HT and those without HT [59]. Other authors [60] confirmed an obvious relationship between HT and PTMC and that HT represents an important factor risk for PTMC in younger adults.

Moreover, it is sufficiently accepted that a relationship also exists between HT and DTC in children, but it is not clear that HT predisposes patients to malignity. In a study on children and adolescents with the association of HT and PC, it has been observed that the number of affected patients is increasing compared to the normal population [61]. Moreover, despite the fact that the mechanism of this association is not clear, children with HT should be classified as being at high risk of cancer [62,63]. In other studies, children and adolescents with HT presented more frequently invasive DTC and familial PC with respect to patients without HT, and a better PC outcome was not observed whether HT was present or not [64]. However, in an elevated number of children and adolescents, HT seemed to be associated with a high risk of developing thyroid nodules but not cancer [65].

Furthermore, the immune response induced by antithyroid antibodies, AbTg, and AbTPO has been considered to be associated with the development and prognosis of PC in patients with HT, and many studies have been carried out on this subject; however, many more will be necessary to try to clarify the role of autoimmune inflammation on PC.

In the present study, despite the absence of the numerous risk factors at the surgery of the primary tumor as above and the presence of AbTg and/or AbTPO in 82.5% of PC/HT patients, metastases developed in 15.5% of cases with significantly higher levels of antibodies with respect to PC/HT patients who did not undergo metastases. Thus, no protective effect on carcinomas by antibodies might be hypothesized with a less favorable prognosis.

Other authors also confirmed the absence of the HT protective effect on PC patients by antithyroid antibodies; they observed that a high AbTPO level correlated with aggressive features, such as larger tumor size, multicentricity, and multifocality, and it was an independent risk factor for tumor recurrence [66]. Other authors observed that AbTg was an independent risk factor for PC with a positive correlation between AbTg and lymph node metastases [67]. Multivariate analysis for metastases of the central compartment in different studies suggested that antibody status, male sex, and tumor size resulted in independent risk factors [68]. Moreover, positive antibody status seemed to be a risk factor for metastatic cervical lymph nodes but a protective factor for distant metastases, hypothesizing that a systemic immunosuppression status may exist in PC patients with distant metastases [69]. On the contrary, other authors reported that high levels of AbTPO appeared to protect against DTC in patients with HT [70], and they indicated that previous autoimmune disease and thyroid autoantibodies exert a favorable influence on the outcome of DTC patients [71]. For further confirmation of these latter data, less cancer recurrence has been reported in PC patients with positive AbTPO [72]. Thus, given the current results reported in the literature, the relationship between thyroid autoimmunity and cancer development in the thyroid gland remains controversial.

Finally, further studies in a large number of PC patients with HT can be useful to explain tumor aggressiveness in some cases like those observed in the present study. In these cases, in the absence of other risk factors, among which is also high values of TSH levels, HT alone seems to represent the most responsible factor, but with a mechanism still to be clarified. For this purpose, in PC patients with HT, it would be important to evaluate specific genetic characteristics that can provide information on cancer progression in this condition.

Some limitations should be examined that are due in part to the retrospective nature of this study since not all the information can be obtained. However, in the present study, these limits did not appear to interfere significantly with the analysis of the data.

Moreover, the study interests a single center, and thus the number of patients with HT is not elevated. Multicentric studies are necessary to obtain a higher number of cases.

Metastasis can return a negative result at the first exam given the slow growth of PC, while it can be ascertained in a late stage. Consequentially, a long-term follow-up is necessary since it could happen that, in some patients, metastases develop later. However, in the present study, the patients were followed for a long period; however, it cannot be excluded that the follow-up period is not long enough to ascertain when an occult metastasis can become evident at the clinical exam. On the other hand, thyroglobulin levels can also be undetectable also in the presence of metastasis.

This study lacks information on genetic alterations, which may affect patient outcomes.

The lack of histopathological findings of some neoplastic lesions was due to the difficulty of locating the site of the metastases. In these conditions, only through the data obtained during the follow-up and monitored over time by clinical exams, radiologic and nuclear medicine procedures, and thyroglobulin sequential changes, could the nature of the neoplastic lesions be validated.

## 5. Conclusions

The present study seems to demonstrate that HT is not a cancer protective factor in PC patients since a less favorable outcome during the follow-up after thyroidectomy and radioiodine ablation was observed with a significant difference compared to patients with PC without HT, in whom DFS was also significantly reduced. These results, confirmed by some authors, but in contrast to those obtained by others, seem to prove that HT alone may represent an important predictor factor for a significantly increased risk of developing metastases over time. This result takes greater importance since in the surgery of the primary tumor, other risk factors were not verified. The data observed in the present PC/HT series could suggest that the chronic autoimmune inflammation and high levels of AbTg and AbTPO seem more correlated with aggressive PC progression rather than to represent a protective factor. However, considering the many conflicting findings on this issue, further studies on larger casuistries, possibly prospective, also evaluating genetic aspects are needed to clarify the different pathophysiological mechanisms in the relationship between HT and PC. Finally, careful observation and follow-up of patients with HT associated with PC is recommended, including, in the close control, the microcarcinomas, in particular those with size >5 mm that were ascertained in large numbers in the present series of patients who developed metastases.

## Figures and Tables

**Table 1:** Demographic and histological characteristics of 97 patients affected by papillary carcinoma (PC) with associated Hashimoto’s thyroiditis (HT) and 206 patients with PC without HT, as controls, at the surgery of the primary tumor.

		PC Patients with HT	PC Patients without HT as Controls	*p*-Value
		(97 Cases)	(206 Cases)	
**Age (years)**		<55 yrs - 59 (60.8%)	<55 yrs - 127 (61.7%)	0.89
		≥55 yrs - 38 (39.2%)	≥55 yrs - 79 (38.3%)	
**Age (years), mean ± SD**		49.1 ± 12.9 (*median* 51)	49.9 ± 14.3 (*median* 50.5)	0.64
**Sex (F/M)**		81 (83.5%)/16 (16.5%)	168 (81.6%)/38 (18.4%)	0.68
**Histology**		Classic variant 88 (90.7%)	Classic variant 179 (86.9%)	0.34
		Follicular variant 9 (9.3%)	Follicular variant 27 (13.1%)	
**Tumor size (mm)**		≤10 mm - 64 (66%)	≤10 mm - 122 (59.2%)	0.26
		>10 mm - 33 (34%)	>10 mm - 84 (40.8%)	
**Tumor size (mm), mean ± SD**		10.1 ± 8.3 (*median* 8)	11.1 ± 8.9 (*median* 10)	0.35
**SN/MNG**		75 (77.3%)/22 (22.7%)	172 (83.5%)/34 (16.5%)	0.20
**TNM**				
T1a N0 M0		64 (66.0%)	122 (59.2%)	0.26
T1b N0 M0		25 (25.8%)	67 (32.5%)	0.23
T2 N0 M0		8 (8.2%)	14 (6.8%)	0.65
T3a N0 M0		0	3 (1.5%)	0.55
Risk stratification *				
High risk	(H)	0	3 (1.5%)	0.23
Low risk	(L)	33 (34%)	81 (39.3%)	0.37
Very low risk	(VL)	64 (66%)	122 (59.2%)	0.26

* European Cancer Task Force classification [33].

**Table 2:** Distribution of AbTg and/or AbTPO in the 97 patients of group 1 at the surgery of the primary tumor.

Antithyroid Antibodies		
AbTg and AbTPO (≤cut/off)		17/97 (17.5%)
AbTg and/or AbTPO (>cut/off)		80/97 (82.5%)
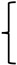	AbTg and AbTPO (>cut/off)	10/80 (12.5%)
	AbTg (>cut/off)	34/80 (42.5%)
	AbTPO (>cut/off)	36/80 (45.0%)

**Table 3:** Characteristics of PC/HT patients at the surgery of the primary tumor and during long-term follow-up.

	At Surgery									At Follow-Up			
Patients n.	Age	Sex	Size (mm)	Histology	TNM	Risk	Tg	AbTg	AbTPO	Metastasis	Tg	AbTg	AbTPO
							(ng/mL)	(IU/mL)	(IU/mL)		(ng/mL)	(IU/mL)	(IU/mL)
**1**	55	F	13	PC	T1b N0M0	L	und	86.4	36.0	1 LN PT	2.1	und	und
**2**	46	M	40	PCFV	T2 N0M0	L	2.7	111.8	7.9	1 LN PT	und	178.0	und
**3**	58	F	8	PC	T1a N0M0	VL	und	153.6	663.0	1 LN PT	und	25.5	12.0
**4**	51	F	5	PC	T1a N0M0	VL	und	163.4	22.0	1 LN PT	1.3	8.3	12.0
**5**	45	F	7	PC	T1a N0M0	VL	1.1	500.0	91.0	1 LN LTC + 1 LN SM	2.0	63.0	21.0
**6**	34	F	10	PC	T1a N0M0	VL	8.9	3.0	80.0	1 LN SV	5.1	5.0	und
**7**	33	F	16	PC	T1b N0M0	L	und	12,419.0	und	1 LN LTC	und	822.0	und
**8**	59	F	11	PC	T1b N0M0	L	50.0	30.0	66.0	1 LN SV	0.5	14.6	und
**9**	54	F	4	PC	T1a N0M0	VL	und	90.1	54.0	1 LN SM	4.8	und	und
**10**	33	F	10	PC	T1a N0M0	VL	und	263.0	404.0	1 LN PT	0.4	412.0	93.2
**11**	41	F	6	PC	T1a N0M0	VL	und	746.5	und	1 LN SM	und	200.0	und
**12**	16	M	12	PC	T1b N0M0	L	und	194.2	98.0	1 LN SM	und	569.0	und
**13**	49	F	25	PC	T2 N0M0	L	und	1969.0	57.0	1 LN LTC	0.7	148.0	29.3
**14**	64	F	20	PC	T1b N0M0	L	0.4	15.6	273.0	1 LN SM	2.3	und	72.0
**15**	27	F	7	PCFV	T1a N0M0	VL	und	und	38.0	1 LN LTC	3.4	50.0	und

PC: papillary carcinoma; PCFV: papillary carcinoma follicular variant; L: low risk; VL: very low risk; Tg: thyroglobulin; AbTg: antithyroglobulin antibodies; AbTPO: thyroid peroxidase antibodies; und: undetectable; LN: lymph node; LTC: laterocervical LN metastasis; SM: submandibular LN metastasis; PT: paratracheal LN metastasis; SV: supraclavicular LN metastasis.

**Table 4:** Characteristics of 16 control PC patients without HT who developed metastases (group 2a) at the surgery of the primary tumor and during long-term follow-up.

	At Surgery									At Follow-Up			
Patients n.	Age	Sex	Size (mm)	Histology	TNM	Risk	Tg	AbTg	AbTPO	Metastasis	Tg	AbTg	AbTPO
							(ng/mL)	(IU/mL)	(IU/mL)		(ng/mL)	(IU/mL)	(IU/mL)
**1**	60	F	15	PC	T1b N0M0	L	0.4	und	und	2 LN LTC	6.4	1	und
**2**	52	F	3	PCFV	T1a N0M0	VL	0.9	und	und	1 LN SM	und	und	und
**3**	74	F	4	PC	T1a N0M0	VL	0.4	10.9	und	1 LN LTC + 1 lung + 1 bone	15.0	4.5	und
**4**	61	F	8	PC	T1a N0M0	VL	4.1	8.6	und	1 LN SM + 1 LN SV	6.0	1.5	und
**5**	34	F	15	PC	T1b N0M0	L	1.9	10.3	10.6	1 LN LTC	2.0	2.0	und
**6**	40	F	20	PC	T1b N0M0	L	1.3	3.4	4.1	1 LN PT	und	5.0	und
**7**	54	M	11	PC	T1b N0M0	L	4.3	10.5	und	1 LN PT	2.3	und	und
**8**	62	F	8	PC	T1a N0M0	VL	18.2	1.1	und	1 LN PT + 1 LN LTC	8.0	und	und
**9**	28	F	6	PC	T1a N0M0	VL	1.0	9.7	und	1 LN LTC	2.7	7.1	und
**10**	45	F	6	PC	T1a N0M0	VL	1.0	und	und	1 LN SM	und	und	und
**11**	34	F	12	PC	T1b N0M0	L	2.4	2.6	und	1 LN LTC	1.0	und	und
**12**	42	F	12	PC	T1b N0M0	L	und	und	6.6	1 LN LTC	und	2.7	2.9
**13**	38	F	15	PC	T1b N0M0	L	und	7.9	4.2	1 LN PT	0.6	und	und
**14**	46	F	7	PC	T1a N0M0	VL	76.1	15.0	und	1 LN SV	3.5	und	und
**15**	64	F	2	PC	T1a N0M0	VL	11.3	und	und	1 LN SM	4.2	und	und
**16**	20	M	20	PC	T1b N0M0	L	1.8	und	und	1 LN PT	2.1	17.6	und

PC: papillary carcinoma; PCFV: papillary carcinoma follicular variant; L: low risk; VL: very low risk; Tg: thyroglobulin; AbTg: antithyroglobulin antibodies; AbTPO: thyroid peroxidase antibodies; und: undetectable; LN: lymph node; LTC: laterocervical LN metastasis; SM: submandibular LN metastasis; PT: paratracheal LN metastasis; SV: supraclavicular LN metastasis; lung: lung metastasis; bone: bone metastasis.

**Table 5:** The univariate logistic regression shows that PC/HT patients had a 2.17-fold higher risk of developing metastasis than PC control patients without HT (*p* = 0.043).

	OR (95%CI)	*p*-Value
**Age (years)**	0.98 (0.95–1.01)	0.105
**Age < 55 years**	1.61 (0.71–3.63)	0.252
**Female gender**	1.52 (0.51–4.54)	0.453
**PC follicular variant**	0.78 (0.22–2.69)	0.690
**Tumor size (mm)**	1.01 (0.97–1.05)	0.720
**Microcarcinoma**	0.64 (0.30–135)	0.241
**HT**	2.17 (1.03–4.60)	0.043

## Data Availability

The data that have been presented in this study are available on reasonable request from the corresponding author.
